# Genetic variation in KSHV encoded microRNAs affects microRNA expression and is associated with multicentric Castleman’s disease risk

**DOI:** 10.1186/1750-9378-7-S1-O5

**Published:** 2012-04-19

**Authors:** Vickie Marshall, Alex Ray, Soo-Jin Han, Thomas Uldrick, Eugene Barsov, Octavio Quinones, Robert Leighty, Nazzarena Labo, Kathy Wyvill, Karen Aleman, Mark N Polizzotto, Richard F Little, David Ott, Robert Yarchoan, Rolf Renne, Denise Whitby

**Affiliations:** 1 Viral Oncology Section, AIDS and Cancer Virus Program, SAIC-Frederick, National Cancer Institute (NCI)-Frederick, Frederick, MD, USA; 2 Department of Molecular Genetics and Microbiology, University of Florida, Gainesville, FL, USA; 3 HIV and AIDS Malignancy Branch, NCI, Bethesda, MD, USA; 4 Retrovirus Assembly Laboratory, AIDS and Cancer Virus Program, SAIC-Frederick, Frederick, MD, USA; 5 Data Management Services, Inc, NCI-Frederick, Frederick, MD, USA

## 

Kaposi sarcoma-associated herpesvirus (KSHV) encodes 12 pre-microRNAs which potentially yield 25 mature microRNAs and have been shown to play prominent roles in the viral lifecycle including maintaining viral latency, evading the host immune response, and controlling lytic replication. We previously reported phylogenetic analysis of the microRNA-coding region of KSHV from Kaposi sarcoma (KS), primary effusion lymphoma (PEL), and multicentric Castleman disease (MCD) patients. We showed a high level of conservation for most sequences, but also a divergent cluster of 5 KSHV sequences including 2 from MCD patients [1]. We additionally observed single nucleotide polymorphisms (SNP) in the sequence of KSHV encoded mature and pre-miRNAs from clinical samples including a SNP in mir-K12-5 reported to result in increased expression of the mature miRNA [2].

To determine whether SNPs in other KSHV encoded miRNAs resulted in differences in miRNA processing and expression we used four complimentary approaches. Analysis of KSHV miRNA expression levels in PEL cell lines using custom ABI real time qPCR assays showed differential expression that correlates with sequence. Lentiviral vectors constructed to express wild type and variant pre-miRNAs were transduced into 293T cells to make stably expressing cell lines. miRNA expression was assessed using custom ABI real time qPCR assays. Luciferase reporter assays were performed following transient transfections of each miRNA. In addition, *in vitro* maturation assays were performed to assess differences in Drosha/DGCR8 and Dicer cleavage between wild type and variant pre-miRNAs. Our results indicate that polymorphisms within the pre-miRNA sequence can cause subtle expression differences as in the case of KSHV miR-K12-6 or more profound changes as observed in miR-K12-5. Our data clearly shows that SNPs can affect pre-miRNA processing resulting in changes in mature miRNA expression levels.

To extend our studies on miRNA variation in MCD patients, KSHV miRNA sequences from 23 MCD patients and 7 patients with a newly described KSHV-associated inflammatory cytokine syndrome (KICS) were examined by amplification, cloning, and sequencing of a 646-bp fragment of K12/T0.7 encoding miRNA-K12-10 and miRNA-K12-12 and a 2.8-kbp fragment containing the remaining 10 pre-microRNAs. Phylogenetic analysis showed a distinct variant cluster consisting exclusively of MCD and KICS patients in all trees. Pearson’s chi-squared analysis revealed 40 single nucleotide polymorphisms (SNPs) at various loci were significantly associated with MCD and KICS risk. Additionally, cluster analysis of these SNPs generated several combinations of three SNPs as putative indicators of MCD and KICS risk. Taken together, these findings show that MCD and KICS patients frequently have unusual KSHV microRNA sequences and suggest association between the observed sequence variation and risk of MCD and KICS.
